# F65. NETWORK ANALYSIS OF EMPATHY, SCHIZOTYPY AND AFFECTIVE STATES IN A COLLEGE SAMPLE

**DOI:** 10.1093/schbul/sby017.596

**Published:** 2018-04-01

**Authors:** Yi Wang, Wen-hua Liu, Hai-song Shi, Raymond C K Chan

**Affiliations:** 1 Institute of Psychology, Chinese Academy of Sciences; 2 School of Health Management, Guangzhou Medical University; 3 North China Electric Power University

## Abstract

**Background:**

Although the deficits of empathy in schizophrenia spectrum disorders has been recognized in previous studies, little is known about the associations between empathy and schizotypal traits. In this study, we examined the associations among empathy, schizotypy and affective states using the psychological network analysis in a college sample to better understand the social cognition deficits in schizophrenia.

**Methods:**

College students (n=1486; male = 574, female = 912; mean age=18.8 years; SD=0.85) were recruited and all of them finished self-reported questionnaire capturing empathy (Interpersonal Reactivity Index, IRI; four subscales: perspective taking, empathic concern, fantasy, personal distress), schizotypy (Wisconsin Psychosis Proneness Scales, including social anhedonia, physical anhedonia, magical ideation and perceptual aberration scales) and affective states (Depression, Anxiety and Stress Scale, 21 items). There were significant sex differences on IRI (female > male for all four subscales, ps < 0.01), DASS depression (male > female, p < 0.01) and schizotypal traits (male > female, ps < 0.05) Psychological networks were constructed taking the subscales of measures as nodes and the edges representing the partial correlation between each pair of nodes controlling all other nodes were estimated using Gaussian graphical model in male and female sample, respectively. Also, the centrality indices, including strength, closeness and betweenness were calculated to identify the central nodes in the network.

**Results:**

In males, cognitive empathy (perspective taking and fantasy) showed strong connections with physical anhedonia, while affective empathy (empathic concern) connected with social anhedonia and stress. Personal distress connected with magical ideation and anxiety; fantasy connected with magical ideation. Regarding the centrality, perceptual aberration had the strongest strength, followed by stress; social anhedonia had the highest closeness and betweenness. In females, cognitive empathy (perspective taking and fantasy) showed strong connection with physical anhedonia, affective empathy (empathic concern) connected with social anhedonia. Personal distress connected with anxiety; fantasy connected with magical ideation. Stress showed strongest strength, followed by anxiety and magical ideation; anxiety had highest betweenness; fantasy had highest closeness followed by social anhedonia.

**Discussion:**

In the present study, we found that cognitive empathy was strongly connected with physical anhedonia, while affective empathy connected with social anhedonia, regardless of sex. In addition, our findings suggested different network interactions among empathy, schizotypal traits and affective states between males and females. The perceptual aberration and social anhedonia play a central role in the network of males while stress and anxiety are important in females.

